# A novel *de novo COL1A1* mutation in a Thai boy with osteogenesis imperfecta born to consanguineous parents

**DOI:** 10.1590/1678-4685-GMB-2016-0033

**Published:** 2017-09-21

**Authors:** Siraprapa Tongkobpetch, Noppachart Limpaphayom, Apiruk Sangsin, Thantrira Porntaveetus, Kanya Suphapeetiporn, Vorasuk Shotelersuk

**Affiliations:** 1 Inter-Department Program of Biomedical Sciences, Faculty of Graduate School, Chulalongkorn University, Bangkok, Thailand; 2 Center of Excellence for Medical Genetics, Department of Pediatrics, Faculty of Medicine, Chulalongkorn University, Bangkok, Thailand.; 3 Excellence Center for Medical Genetics, King Chulalongkorn Memorial Hospital, the Thai Red Cross Society, Bangkok, Thailand.; 4 Department of Orthopaedics, Faculty of Medicine, Chulalongkorn University, Bangkok, Thailand.; 5 Department of Orthopedics, Faculty of Medicine, Chiang Mai University, Chiang Mai, Thailand.; 6 Department of Physiology, Faculty of Dentistry, Chulalongkorn University, Bangkok, Thailand.; 7 STAR on Craniofacial and Skeletal Disorders, Chulalongkorn University, Bangkok, Thailand.

**Keywords:** osteogenesis imperfect, COL1A1, exome sequencing, next generation sequencing, Thai

## Abstract

Osteogenesis imperfecta (OI) is genetically heterogeneous. Mutations in *COL1A1* and *COL1A2* are responsible for at least 90% of the cases, which are transmitted in an autosomal dominant manner or are *de novo* events. We identified a Thai boy with OI whose parents were first cousins. Because the proband was the product of a consanguineous marriage, we hypothesized that he might be homozygous for a mutation in a known gene causing a recessive form of OI. Using whole exome sequencing (WES), we did not find any pathogenic mutations in any known gene responsible for an autosomal recessive form of OI. Instead, we identified a *COL1A1* frameshift mutation, c.1290delG (p.Gly431Valfs*110) in heterozygosis. By Sanger sequencing, the mutation was confirmed in the proband, and not detected in his parents, indicating that it was a *de novo* mutation. These findings had implication for genetic counseling. In conclusion, we expanded the mutational spectrum of *COL1A1* and provided another example of a *de novo* pathogenic mutation in heterozygosis in a patient born to consanguineous parents.

Osteogenesis imperfecta (OI), or brittle bone disease, is a disorder characterized by low bone mass, bone fragility and often short stature. Extraskeletal manifestations may include blue sclerae, hearing loss and dentinogenesis imperfecta. The phenotypic severity of OI ranges from a very mild form without fractures to intrauterine fractures and perinatal lethality. The prevalence of OI is estimated to be about 7 per 100,000 births (Lindahl *et al.*, 2015; Stevenson *et al.*, 2012)

OI is genetically heterogeneous. About 90% of OI patients carry mutations in the *COL1A1* or *COL1A2* genes, which are *de novo* events or transmitted in an autosomal dominant manner due to parental mosaicism. Other inherited forms are rare and can be caused by mutations in different genes, including one gene responsible for the dominant form, *IFITM5*, 14 genes for the recessive form (*BMP1, CRTAP, CREB3L1, FKBP10, LEPRE1, PLOD2, PPIB, SEC24D, SERPINF1, SERPINH1, SP7, SPARC, TMEM38B* and *WNT1* (Forlino and Marini, 2016; Garbes *et al.*, 2015; Mendoza-Londono *et al.*, 2015), and one gene for the X-linked form, *MBTPS2* (Lindert *et al.*, 2016). These genes play an important role in the process of collagen type I synthesis or in the control of osteoblast differentiation or function. Notably, the causative mutation in some patients with OI remains unidentified. Identification of mutations is important for genetic counseling, risk assessment, and facilitation of prenatal or preimplantation diagnosis.

Since OI can be caused by mutations in at least 18 genes, Sanger sequencing of all these genes would be time-consuming and prohibitively expensive. In 2010, whole exome sequencing (WES) was used to successfully discover a new gene for a genetic disease for the first time (Ng *et al.*, 2010). Several novel causative genes were subsequently reported by next generation sequencing (NGS), including the most recently identified X-linked gene for OI, *MBTPS2* (Lindert *et al.*, 2016). NGS can analyze multiple regions in one reaction. It is therefore, a suitable tool for studying diseases with genetic heterogeneity, such as OI.

This study reports the clinical features and mutation analysis of a child with OI. He is the fourth child born to a healthy couple of first cousins. Their three older brothers are healthy, and there is no family history of OI or other bone diseases. He was born at full term and delivered by cesarean section, because of breech presentation. His birth weight was 2,950 g (25^th^ centile) and his birth length was 47 cm (3^rd^ centile). At birth, he had a closed fracture of the right femoral shaft, and was treated with a long leg cast for two weeks. His second fracture occurred at the age of one month at the left proximal femoral shaft. Serum calcium and alkaline phosphatase were within normal limit. DEXA scan showed markedly decreased bone density (lumbar spine 0.159 g/cm^2^, left hip 0.163 g/cm^2^, whole body 0.262 g/cm^2^). OI was then diagnosed clinically. Pamidronate was started at the age of five months.

From birth to the age of three years, his height was between the 3^rd^ and 10^th^ centiles, weight below the 3^rd^ centile, and head circumference around the 75^th^ centile, consistent with relative macrocephaly. By the age of four years, even under medication, he had six lower limb fractures caused by minor traumas. Bone deformities of lower extremities became notable. His cranium radiographs revealed Wormian bones, an important characteristic of OI. No clinical or radiographic evidence of spinal or upper extremity fracture was noted. From the age of four to eight years, he was free from fractures. However, at the age of eight years, he had a fracture of the right femoral shaft after a simple fall. His mental development was appropriate. At the last follow up, when he was 11 years old, his height was 123 cm (Z score of −3) and his weight was 27 kg (Z score of −1). He also had blue sclerae and pectus carinatum. Dentinogenesis imperfecta was not observed (Figure 1A). He had a limping gait due to leg length discrepancy. The standing anteroposteriorradiograph of the pelvis and femora revealed tilting of pelvis and malunion at the right femoral shaft causing varus deformity (Figure 1B). There was a hypertrophic nonunion of the shaft of the right femur (Figure 1B, C). Multiple dense metaphyseal lines caused by bisphosphonate therapy were notable in both distal femoral and proximal tibia metaphyses. He did not show platyspondyly or scoliosis. Based on clinical findings and follow-up, as well as radiological findings, the diagnosis of OI type IV was given to the patient.

**Figure 1:**
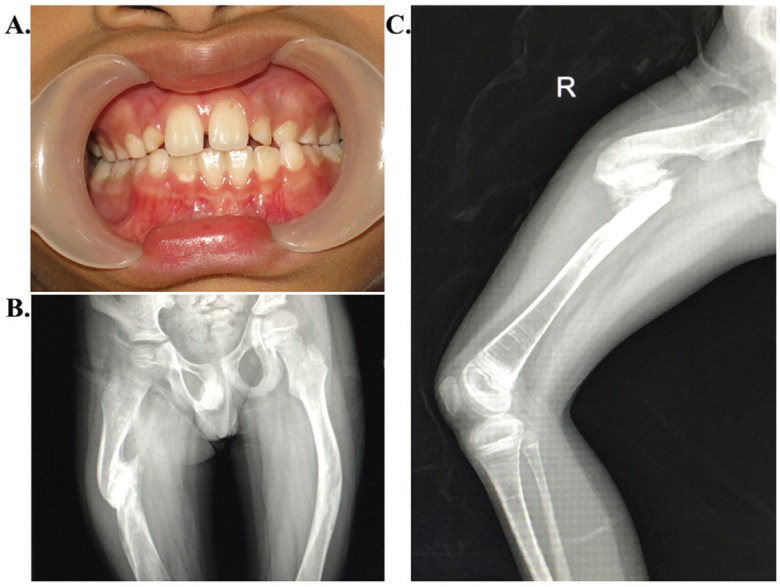
Clinical and radiographic features of the patient with osteogenesis imperfecta. (A) Dentinogenesis imperfecta was not present. (B) Standing anteroposterior radiograph of the pelvis and femora showing tilting of the pelvis and varus deformity of both femora. (C) Radiograph of his right femur showing a hypertrophic malunion of the femoral shaft.

After informed consent was obtained, genomic DNA was isolated from peripheral blood leukocytes using a Puregene Blood kit (Qiagen, Hilden, Germany). The genomic DNA was sent to Macrogen Inc. (Seoul, South Korea), for whole-exome sequencing (WES). DNA was captured using the TruSeq DNA Sample Prep kit (Agilent Technologies, Santa Clara, CA) and sequenced on a Hiseq2000 instrument. Base calling was performed and quality scores analyzed using Real Time Analysis software version 1.7. Sequence reads were aligned against the University of California Santa Cruz human genome assembly hg19 using Burrows-Wheeler Alignment software (BWA). Single-nucleotide variants (SNVs) and insertions/deletions (Indels) were detected by Sequence Alignment/Map tools (SAMtools) and annotated against dbSNP & the 1000 Genomes Project. After quality filtering, we looked for variants located in the coding regions of known skeletal dysplasia genes for all potential pathogenic SNVs and Indels. Variant calling exclusion criteria were: (a) coverage < 10×, (b) quality score < 20, (c) minor allele frequency ≥1% in the 1000 Genomes Project, and (d) non-coding variants and synonymous exonic variants. The remaining variants were subsequently filtered out if they were present in our in-house database of 200 unrelated Thai exomes. Existing SNVs or known pathogenic mutations were filtered out using the Human Gene Mutation Database (HGMD) and the Exome Aggregation Consortium database (ExAC). The pathogenic variant identified was confirmed by Sanger sequencing. DNA samples from the unaffected parents and one of the unaffected brother of the patient were also Sanger sequenced to search for the mutation.

With a history of parental consanguinity, we hypothesized that our patient could be homozygous for a mutation in one of the 14 genes responsible for an autosomal recessive form of OI. A caveat is that he manifested blue sclerae. Patients with recessive forms of OI generally do not have blue sclerae or other secondary features. However, this is not absolute, as there have been reports of patients with recessive forms of OI who had grayish sclerae (Becker *et al.*, 2011). Nonetheless, no mutations in any one of the known genes responsible for an autosomal recessive form of OI was identified in our patient.

We then looked for a heterozygous mutation for the autosomal dominant form of OI, and found a c.1290delG mutation in exon 19 of the *COL1A1* gene. The deletion leads to a frameshift, resulting in a premature termination codon (PTC) at residue 540 (p.Gly431Valfs*110). The gene product, the alpha 1 chain of type I collagen, is expected to be shortened from 1464 to 539 amino acid residues. Sanger sequencing confirmed the presence of the *COL1A1* c.1290delG mutation in heterozygosis (Figure 2). It was not present in neither of his parents nor the unaffected brother, indicating a *de novo* mutation. This mutation was not present in the HGMD, the Osteogenesis Imperfecta Variant Database, or the in-house 200 Thai Exome Database.

**Figure 2:**
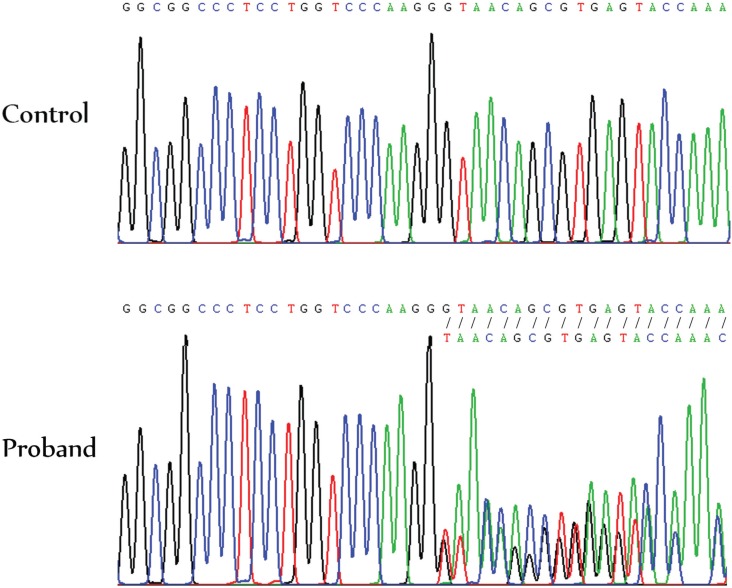
Electropherogram showing the *COL1A1* c.1290delG mutation in heterozygosis in the proband compared to a normal control.

Dentinogenesis imperfecta was not present in our patient. It has been suggested that collagen plays non-identical roles in bone and dentin, since individuals with *COL1A1* mutations have a wide variety of dentin and bone defects (O’Connell and Marini, 1999; Rauch *et al.*, 2010).

Mutations in the *COL1A1* gene can lead to OI by either haploinsufficiency or dominant negative effects. Haploinsufficiency is usually the consequence of splice site mutations, nonsense mutations, deletions or insertions. These mutations usually create a premature termination codon. These aberrant RNAs would usually be degraded by nonsense-mediated mRNA decay (NMD). With normal alpha chains of type I collagen being produced from the wild-type allele, haploinsufficiency generally leads to a mild OI phenotype (Marini *et al.*, 2007). On the other hand, dominant negative effects result from missense mutations or from premature termination codon mutations that avoid NMD; the mutated alpha chain binding to normal alpha chains produced by the wild-type allele results in abnormal collagen type I. Classically known OI mutations with dominant-negative effect are the substitution of an amino acid for one of the obligatory glycine residues occurring in every third position along the chain of COL1A1, and other mutations, such as exon skipping defects or in-frame deletions. Most cases of OI with dominant negative effects are typically more severe than those with haploinsufficiency (Ben Amor *et al.*, 2013).

Rauch *et al.* (2010) reported that mean Z-score for height was −1.3 in patients harboring mutations that lead to haploinsufficiency, and −5.5, for those with helical mutations, causing a dominant-negative effect. The patient presented here had Z-scores of height and weight of ≤ −3.0, which were between these two groups. Since the cyclical intravenous pamidronate treatment was started in this patient at the age of five months, the Z-score for height might have been less than −3 at the age of 11, if he had not received the medication. In fact, early treatment with intravenous disodium pamidronate may prevent scoliosis and basilar impression (Astrom *et al.*, 2007), and four years of cyclical intravenous pamidronate treatment was reported to lead to significant height gains in moderately to severely affected OI patients (Zeitlin *et al.*, 2003). Taken together, the z-scores of our patient's height and weight point to the form caused by a dominant negative effect (Rauch *et al.*, 2010).

The severe phenotype of our patient suggested that the *COL1A1* c.1290delG mutation led to a truncated alpha chain able to bind to the normal alpha chains, resulting in the production of defective collagen type I. The more severe phenotype of the patient reported could be explained by a possible abnormality in NMD efficiency. Although the mutation found in our patient is located in the NMD sensitive zone, 50-55 nucleotides upstream of the last exon-exon junction, the mutant RNA might still escape NMD. In 2014 the evidence pointing to the inter-individual variability in NMD efficiency and its correlation with clinical presentations was reviewed, and it was proposed that it was a common phenomenon in human populations (Nguyen *et al.*, 2014). Therefore, it is possible that the mutant RNA escapes NMD, at least partially, in our patient. There were previous reports of patients with severe OI who had premature truncation mutations. A Korean patient with OI type IV had an eight-base pair deletion in exon 46 (Lee *et al.*, 2006). Vietnamese patients with OI type III or IV were reported to have frameshift mutations located in the NMD sensitive zone, similar to our patient (Ho Duy *et al.*, 2016). Taken together, we hypothesize that variable NMD efficiency may be a cause of variable OI phenotypes in different patients with similar out-of-frame mutations.

Identification of this pathogenic mutation allowed more accurate genetic counseling, ruling out recessive inheritance. Although the recurrence risk in this case is likely to be very low, the probability of parental mosaicism had to be considered, since no other tissue than blood was tested. An empirical recurrence risk of 27% for the perinatal lethal form of OI (type II) in the offspring of parents carrying mosaicism for collagen I mutations was reported (Pyott *et al.*, 2011). With mutation identification, prenatal or preimplantation diagnosis can be provided.

In this study, using WES, we successfully identified a novel *de novo* mutation in the *COL1A1* gene in a patient with OI, expanding its mutational spectrum. In addition, we provided another example of identification of a heterozygous *de novo* mutation as the cause of a disease in a patient born to consanguineous parents, which allowed more accurate genetic counseling.
